# Impact of Appointment-Based Medication Synchronization on Proportion of Days Covered for Chronic Medications

**DOI:** 10.3390/pharmacy6020044

**Published:** 2018-05-22

**Authors:** Nancy Dao, Sun Lee, Micah Hata, Lord Sarino

**Affiliations:** 1 Western University of Health Sciences College of Pharmacy, Pomona, CA 91766, USA; slee22@westernu.edu (S.L.); mhata@westernu.edu (M.H.); 2 Ralphs Grocery Company, Compton, CA 92620, USA; lord.sarino@ralphs.com

**Keywords:** appointment-based model, adherence, community pharmacy, medication synchronization, proportion of days covered

## Abstract

Appointment-based medication synchronization (ABMS) programs have been associated with increased adherence and persistence to chronic medications. Adherence to statin therapy, angiotensin-converting enzyme inhibitors (ACEIs), angiotensin receptor blockers (ARBs), and non-insulin antidiabetic medications (NIDM) are used to determine a health plan’s Centers for Medicare and Medicaid Services (CMS) Star Rating under a pay-for-performance model. The objective of this study was to evaluate the impact of implementing an ABMS program on overall pharmacy adherence measures for statins, ACEI/ARBs, and NIDM, as presented through the Electronic Quality Improvement Platform for Plans and Pharmacies (EQuIPP©) platform. This retrospective, pre-post ABMS program study evaluated EQuIPP© generated adherence performance measures, represented as proportion of days covered (PDC), 6-months before and 6- and 12-months after the ABMS service for statin therapy, ACEIs/ARBs, and NIDM. All adherence measures showed statistically significant improvement in PDC percentage post ABMS implementation, except for NIDM percentage in 6-months post-ABMS service. This study shows that a comprehensive medication synchronization program can enhance adherence measures that are important to health plans to increase CMS Star Rating under a pay-for-performance model.

## 1. Introduction

As the United States health system moves toward a pay-for-performance model, reimbursement for health care-related services are based on predefined outcomes that aim to reflect the quality of care provided. A pay-for-performance model incentivizes providers to improve health outcomes for patients while maximizing cost-savings. Under a pay-for-performance model, health plans are rated based on Centers for Medicare and Medicaid Services (CMS) Star Rating criteria. Some CMS Star Rating criteria are directly related to medication adherence, such as adherence to statin therapy, angiotensin-converting enzyme inhibitors (ACEIs), angiotensin receptor blockers (ARBs), and non-insulin antidiabetic medications (NIDM) [1].

Medication nonadherence in chronic disease such as diabetes and hypertension contribute to increased morbidity, mortality, and unnecessary health care costs [2,3,4]. There are multiple factors affecting medication nonadherence such as socioeconomic, patient-related, condition-related, therapy-related, and health system-related factors. An appointment-based medication synchronization (ABMS) program allows a pharmacist a scheduled opportunity to build relationships with their patients to identify patient-related factors affecting adherence such as patient attitudes, beliefs, and perceptions about their medications and health. In addition, therapy-related and condition-related factors can be addressed by reducing complexity of drug regimens, managing side effects, and communicating disease progression to providers for enhanced disease state management. By synchronizing multiple medications to be filled at one day of the month, the pharmacy can address multiple barriers that affect medication adherence [5,6].

Medication synchronization programs have been associated with increased adherence and persistence to chronic medications [5,7]. Both chain and independent community pharmacies offer medication synchronization services (e.g., MedSync, Sync Your Meds) [8]. Ralphs Pharmacy© has been offering the medication synchronization program through an appointment-based medication synchronization (ABMS) model since 2014. Prior to the implementation of ABMS programs, pharmacies offered automatic refill prescription services that refilled medications based on the last fill date. Two fundamental differences exist between an ABMS program and an automatic refill prescription service [8,9]:

1. Prescription refills for an ABMS patient are synchronized to be completed on a single day of the month.
2. The pharmacist regularly engages an ABMS patient in monthly phone calls to confirm which medications are to be filled prior to the scheduled prescription pick up.

These differences allow for pharmacists to proactively fill prescriptions by refilling them days prior to a monthly pick up date specified by the patient, identify medication therapy changes or issues (e.g., nonadherence, adverse event management, appropriateness of therapy), increase patient satisfaction with pharmacy services, and reduce the number of visits needed to the pharmacy [10,11].

The objective of this study was to evaluate the impact of implementing an ABMS program on pharmacy adherence measures for statins, ACEI/ARBs, and non-insulin diabetic medications (NIDM). The Electronic Quality Improvement Platform for Plans and Pharmacies (EQuIPP©) is a platform that allows the pharmacy to analyze their impact on patient adherence measures. EQuIPP© provides performance data that is unbiased and reflects current CMS Star Ratings [12]. It was hypothesized that initiation of ABMS would increase EQuIPP© adherence measures for these drug classes.

## 2. Materials and Methods

### 2.1. Appointment-Based Medication Synchronization (ABMS) Protocol

This study was conducted for Ralphs Pharmacy© within the Ralphs Grocery Company network. Ralphs Pharmacy© is a Kroger Company owned grocery store pharmacy spanning Southern California. There are 193 Ralphs Grocery Stores locations of which 79 locations provide pharmacy services. The ABMS program was officially started at Ralphs in April 2014; however, individual Ralphs pharmacies implemented the program at different dates resulting in varying “initial enrollment” dates for each store. By September 2016, all Ralphs locations had implemented the ABMS program. The program has been advertised by Ralphs Pharmacy© staff members to all patients with chronic medications. The ABMS program was an ‘opt-in’ service where the pharmacist was in charge of overseeing and managing the ABMS protocol.

To enroll patients into the ABMS program, it was required that:

- A member of the pharmacy staff explains the ABM program and have the patient sign an enrollment form.
- The patient picks a day of the month when prescriptions are to be ready (sync date).
- The pharmacist performs a comprehensive medication review for the patient (in-person or telephonic), screening for drug-related issues (e.g., adherence, drug-drug interactions, etc.), immunizations, and other pharmacy clinical programs that may benefit the patient (ex. diabetes coaching, naloxone dispensing, hormonal contraception furnishing, etc.) to finalize the prescriptions that will be part of ABMS program.
- Upon patient request, the pharmacy must notify the prescriber of the patient’s ABMS enrollment and the sync date.

Once the patient was enrolled in the ABMS service, the pharmacy offered the following services:

- The pharmacy technician made calls for any sync patients at least 4 days prior to their sync date, asked the patient if there had been any changes to medications that would prompt a pharmacist evaluation, and scheduled the prescriptions.
- If the patient was unable to be reached, the pharmacy technician left a message confirming the monthly order. The patient was offered to pick up medications after 2 PM of their medication-synced date. If necessary, patients were asked to pay an extra co-pay one time for a short fill of each medication to make all refills due on the same day.
- During the monthly calls to the patient, the pharmacist updated any information in regards to doctor’s appointments, hospital/urgent care visits, and changes in health status.
- The pharmacist would counsel patients on all medications as necessary when they picked up their medications and offered any clinical services or immunizations as needed.

After a patient was enrolled in the ABMS program, a paper-based document specific to the patient was kept on file, which contained:

- name of the patient
- sync date
- date of birth
- phone number
- enrollment date into the program
- third party plan information
- list of medications (name and strength)
- immunization history
- a space to designate if the patient is in other medication therapy management platforms, such as OutcomesMTM or Mirixa

This document was continually updated by the pharmacy technicians, with a brand-new sheet being filled out at the start of each calendar year. Two binders were used to alphabetically organize the patient documents based on their sync date. One binder was for patients whose sync date was on the 1st to 15th, and the other binder for sync dates from the 16th to the 31st. The only electronic documentation consisted of selecting an ABMS enrollment designation in the patient profile and recording the date of enrollment in the pharmacy operating system.

### 2.2. Study Design

This retrospective, pre-post ABMS program study evaluated EQuIPP© generated adherence performance measures from 77 Ralphs Pharmacies© located in California between January 2014 and May 2017. Although the ABMS program was offered since April 2014, each pharmacy had different initial service starting dates, as the first patient enrolled in the program was different in each store. ABMS implementation date for each store was identified by recognizing the date for the first fill of an ABMS enrolled prescription. This study received exempt status approval from the Western University of Health Sciences Institutional Review Board.

EQuIPP© PDC percentages were generated monthly as a reflection of the average PDC percentage of the previous 6-month period for each pharmacy. Once each store’s initial ABMS program start date was identified, EQuIPP© PDC percentages from 6 months prior to the ABMS program offered date were collected from each store. Additionally, EQuIPP© PDC percentages from 6 months after, and 12 months after the initial ABMS program start date for each store were also recorded.

### 2.3. Outcome Measures

The EQuIPP© adherence percent measure captures the volume of patients who have a proportion of days covered (PDC) percentage above or equal to 80% in each of the 3 medication classes. This performance benchmark for adherence is represented through EQuIPP© as a percentage (e.g., statin adherence percentage of 83% means that out of all patients who primarily fill their prescription for statins at that pharmacy, 83% of those patients have a PDC % ≥80%). The pharmacy which fills the most prescriptions claims within the targeted drug class for a specific patient within the 6-month calendar range will have that patient count towards their adherence percentages in EQuIPP© and be considered that patient’s primary pharmacy [12]. This EQuIPP© PDC percentage includes all primary patients on a medication in that drug class regardless of ABMS enrollment. Adherence PDC percentage goals for ACEI/ARBs, statin, and NIDM were defined as above or equal to 83%, 82%, and 83%, respectively The EQuIPP© defined PDC percentage goals are a reflection of the 2017 CMS defined PDC thresholds for five-star ratings for Medicare insurance plans [12].

### 2.4. Statistical Analysis

Data were analyzed using SPSS v24.0 (SPSS Inc., Chicago, IL, USA). A paired *t*-test was used to compare the three adherence percentages (statin, ACEi/ARB, NIDM) 6-month before and 6- and 12-months after the ABMS service.

## 3. Results

Seventy-seven Ralphs Pharmacies© in and around Southern California were included in the study as there were two pharmacies that did not have patients with the electronic designation of ABMS enrollment for the study period. This was likely due to the staff at these pharmacies incorrectly documenting ABMS enrollment in the patients’ electronic profiles. A total of 8210 patients were enrolled in the ABMS service by the end of the study period as identified by their ABMS program enrollment designation in the pharmacy operating system. Three pharmacies (*n* = 3) were excluded from the 12-month post-ABMS implementation analysis due to initiation of the service after May 2016, where EQuIPP© generated data was not available at the time of data collection, as our study period ended May 2017. There were only EQuIPP© data available starting from January 2014 as Ralphs Pharmacies© was not registered to receive information from EQuIPP© prior to January 2014.

**Figure 1:**
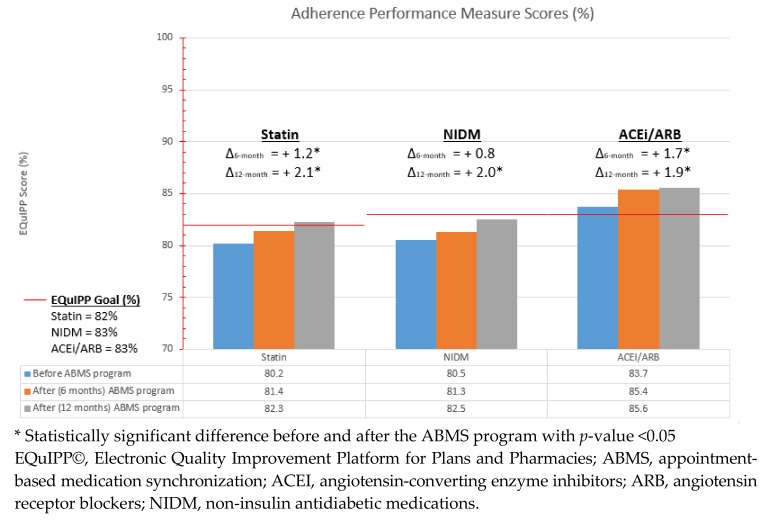
Ralphs Pharmacy© EQuIPP© Adherence Performance Before and After ABMS Implementation.

All outcomes measures showed statistically significant improvement in PDC percentages, except for NIDM percentages in 6-month post ABMS service (Figure 1). Statin adherence 12-months post-ABMS program initiation improved from 80.06% to 82.31% (*p* < 0.01), meeting the EQuIPP© defined PDC percentage of 82% for stores with available EQuIPP© data 12 months after ABMS implementation Average ACEI/ARB adherence was consistently above the EQuIPP© benchmark goal of 83%, with statistically significant improvements in adherence 6- and 12-months after ABMS implementation.

From the 77 Ralphs Pharmacies© included in the study, the pharmacies that did not exceed 80% PDC adherence in EQuIPP© 6 months prior to implementation, were analyzed to determine if adherence measures improved after ABMS implementation. This criteria resulted in a reduction in sample size for statin (*n* = 37), NIDM (*n* = 39), and ACEI/ARB (*n* = 8) measures from the original 77 pharmacies included (Table 1). For the analysis of 12-months post-ABMS implementation for pharmacies with an initial PDC <80%, the sample size was further reduced for statin (*n* = 36) and NIDM (*n* = 37) medication classes due to a lack of EQuIPP© data availability based on ABMS implementation date (Table 2).

For the pharmacies that prior to ABMS did not the PDC percentage goals, there was a statistically significant improvement in all three medication classes six months after implementation, in addition to 12-months post-implementation (Table 1 and Table 2). Despite statistically significant improvements in adherence, these stores 12-months post-implementation of ABMS did not reach EQuIPP©-defined PDC percentage goals (Table 2).

## 4. Discussion

With the adoption of the Appointment-Based Medication Synchronization (ABMS) program across all Ralphs Pharmacies© in California, the overall adherence percentages steadily improved over a 12-month period (Figure 1). The ABMS program could provide a feasible solution to help community pharmacies deliver quality services to customers and help their customers meet optimal therapy outcomes through improved adherence. The EQuIPP© generated PDC percentage benchmarks are based on CMS defined thresholds for a five-star rating for Medicare Part C and D third-party plans. These PDC percentages for the medication classes included in our study became triple-weighted measures for Medicare Part D plans. Medicare Part C plans also have triple-weighted measures associated with disease state control, which could be affected by improved adherence. These measures include percentage of plan members aged 18–75 years with diabetes who had an A1c lab ≤9%, percentage of plan members aged 18–75 years with diabetes whose most recent cholesterol test showed LDL-C <100 mg/dL, and percentage of plan members aged 18–85 years with hypertension whose blood pressure was adequately controlled blood pressure (<140/90 mmHg). Improved adherence of chronic medications could increase the star ratings for these triple-weighted measures for Medicare Part C and Part D plans [13]. Therefore, third party plans have an incentive to focus on improving medication adherence and patient outcomes. A pharmacy’s contribution to improving star ratings through adherence programs can positively contribute to improved star ratings and increase the likelihood of the pharmacy remaining in the preferred pharmacy network for plan members^14^. In addition, various interventions made by community pharmacists through the ABMS program could help improve patient outcomes, which can result in reduced direct and indirect remuneration (DIR) fees [14].

This study demonstrated a statistically significant increase in adherence measures for chronic medication therapy of statin, NIDM, and ACE/ARB over the period of 6- and 12-months post-ABMS implementation. Notably, statin PDC increased from 80.06%, 6 months prior to ABMS, to 82.31%, 12 months post-ABMS implementation, which exceeded the EQUIPP© PDC percentage goal of 82%. These results demonstrate the potential benefit continued ABMS program implementation and enrollment could have on improved adherence. While the increase in PDC percentages was statistically significant, the impact of a 2% improvement in adherence measures on patient clinical outcomes cannot be determined from this study. A low increase in PDC percentages could be related to the number of participating patients in the ABMS program. The number of patients enrolled in the ABMS program during the study period may appear to be low, but it is difficult to determine the percentage of patients in the ABMS program per pharmacy because the number of eligible patients who declined the service was not recorded. Still, there may be a more clinically significant increase in PDC percentages seen with higher patient participation in the ABMS program. Participation, particularly in the early stages of adoption of the program, was dependent on the magnitude of ABMS promotion at each store and was largely affected by pharmacy staff buy-in of the program and difficulties utilizing a paper-based system.

The ABMS program allows prescriptions to be filled in advance five to six days prior to the expected refill dates, similar to other refill programs, which could have a beneficial effect on pharmacy operations. Since pharmacy staff members are aware of which prescriptions need to be filled ahead of time, this should allow pharmacy personnel to effectively allocate time for processing of unexpected acute prescriptions that occur throughout the day. Future studies would need to be conducted to evaluate if rates of prescriptions being returned to the pharmacy stock due to absence of prescription pick up or out of stock medications decrease, since pharmacy staff will have prior knowledge of which medications need to be filled for a subset of patients.

From a patient perspective, a previous study looking at satisfaction rates with an ABMS program at a grocery store pharmacy, showed overall high patient satisfaction. Major themes identified in the study through open-ended statements indicated satisfaction of the program through improved convenience and general appreciation for the program [11]. This study, however, did not assess barriers to initial ABMS enrollment. A potential barrier to enrollment could be financial burden of “short fills” needed prior to receiving all prescriptions on their sync date. Pharmacies must charge a co-pay for these “short fills”, or fills that are dispensed at less than the prescribed quantity, to ensure that the patient arriving to pick up their medications on the sync date is due for a refill for all medications. Future studies would need to identify the major barriers that exist for ABMS enrollment, as previous studies have demonstrated the value of the program to patient adherence and program satisfaction. The results of these studies can possibly give momentum to the motion of pharmacies or third party payers waiving the prorated copay of “short fills” to eliminate this cost barrier to adherence through the ABMS program.

The ABMS program provides an opportunity for the pharmacist to have a more active role in medication therapy management (MTM). The ABMS program structure allows the pharmacy staff to dictate the day when all of a patient’s medications are going to be prepared each month (or every three months). Since the pharmacy staff knows when that day is, they can schedule a recurring patient appointment prior to that day that provides an opportunity for pharmacists to identify and address medication-related needs and improve the patient-pharmacist relationship. An automatic refill program also allows a pharmacy to anticipate when medications need to be prepared for a patient. However, if a patient is on multiple medications and the computer system is automatically refilling them on different days of the month, it is cumbersome and inefficient to have a pharmacist contact the patient each time this is done to identify potential issues related to that patient’s medications. Syncing the patient’s medications to a single fill date creates an opportunity for the pharmacist to have a recurring MTM session with the patient at the same time each month to identify changes to medication therapy (ex. therapy discontinuations or dosing changings), adherence issues, potential adverse drug reactions, recent hospitalizations, and other clinical assessments. During these MTM sessions, pharmacists can more easily determine if the patient would be eligible for other clinical services offered by the pharmacy, such as immunizations. A pharmacist conducting the monthly calls for ABMS patients is better able to identify patients indicated for influenza vaccinations as the vaccine becomes available, and many of the patients can receive the vaccination on their sync date. The pharmacies in our study did not have a way to electronically record patient specific interventions identified through the ABMS program for data collection and analysis. Current literature supports the improvement of patient adherence to chronic medications in association to ABMS, however, further studies need to be conducted to assess its impact on clinical interventions (e.g., immunization rates) and patient health outcomes (e.g., myocardial infarction, stroke).

### Limitations

The research involved several limitations. The retrospective nature of the study did not account for other factors that may have improved adherence during this time, such as improvements in health literacy or use of other adherence tools not provided by the pharmacy (ex. pill box use at home). The EQuIPP© PDC percentage measures are representative of a rolling six-month performance period. Therefore, it is difficult to assess pharmacy adherence performance from month to month. However, during the study period, no new clinical programs to address adherence were implemented on a pharmacy level that would directly affect the PDC percentages. The EQuIPP© PDC percentages reflect percent volume of primary patients of the pharmacy who individually achieved at least 80% adherence. Therefore, this eliminates our ability to interpret if there is an association of improved adherence on the patient level. However, previous studies have demonstrated an improved adherence to chronic medication on a patient level through an ABMS program [4,5,8].

Another limitation is that we were not able to calculate the percentage of ABMS enrolled patients per store to assess the program uptake and factors that may influence participation. Our ability to pull data from the stores for analysis based on the pharmacy staff ability to correctly document enrollment into the electronic patient profile. This documentation into the electronic patient profile was necessary for data collection but was not truly needed for the patient to be receiving the benefits of the ABMS program.

In addition, this analysis did not control for factors such as medication regimen complexity, patient demographics, or degree of patient engagement in their health, which all could potentially influence medication adherence [6]. Although, this study analyzed adherence performance on a pharmacy level, individual patient factors affecting medication adherence are the major contributors to whether a patient will benefit from the ABMS program, which in turn will affect the EQuIPP© adherence measures.

## 5. Conclusions

A comprehensive medication synchronization program can enhance adherence measures that are important to pharmacies and health plans to increase CMS Star Rating under a pay-for-performance model.

## Figures and Tables

**Table 1:** EQuIPP© Adherence Performance Percentages for Pharmacies with Initial PDC <80%, 6 Months After ABMS Implementation.

Medication Class	EQuIPP© PDC Goal	6 Months Prior to Implementation	6 Months Post Implementation	*p* Value
**Statin (*n* = 37)**	82%	76.45	79.18	<0.001
**NIDM (*n* = 39)**	83%	75.88	80.64	<0.001
**ACEI/ARB (*n* = 8)**	83%	76.79	82.73	0.001

Abbreviations used: EQuIPP©, Electronic Quality Improvement Platform for Plans and Pharmacies, PDC, Proportion of days covered, ABMS, appointment-based medication synchronization, ACEI, angiotensin-converting enzyme inhibitors, ARB, angiotensin receptor blockers, NIDM, non-insulin antidiabetic medications.

**Table 2:** EQuIPP© Adherence Performance Percentages for Pharmacies with Initial PDC <80%, 12 Months After ABMS Implementation.

Medication Class	EQuIPP^©^ PDC Goal	6 Months Prior to Implementation	12 Months Post Implementation	*p* Value
**Statin (*n* = 36)**	82%	76.44	80.99	<0.001
**NIDM (*n* = 37)**	83%	76.06	81.39	<0.001
**ACEI/ARB (*n* = 8)**	83%	76.79	81.94	0.002

Abbreviations used: EQuIPP©, Electronic Quality Improvement Platform for Plans and Pharmacies, PDC, Proportion of days covered, ABMS, appointment-based medication synchronization, ACEI, angiotensin-converting enzyme inhibitors, ARB, angiotensin receptor blockers, NIDM, non-insulin antidiabetic medications.
